# Frank Abbott, M.D.

**Published:** 1897-06

**Authors:** 


### Obituary.

### FRANK ABBOTT, M.D.

Dr. Frank Abbott's sudden death on April 20, and noticed briefly in our last issue, has occasioned many expressions of profound regret throughout the dental profession. Few men were better known, for he has been an active and positive factor in dental work ever since he began its practice in New York City. His influence has been extended, and whatever place he may occupy in the future history of dentistry, it can never be truly written without his work filling in it a very prominent position.

Dr. Abbott's positive nature led him to advocate his views with an earnestness that oftentimes, if they failed to carry conviction, engendered respect for the man, for it was felt he battled conscientiously for the opinions entertained, and that without regard to the opposition aroused. With it all, he was genial in companionship, ever ready to help where help was most needed, and throughout loyal to his profession and its work.

This is not the time or place to analyze his labor as an author or investigator. His writings, in connection with the late Dr. Heitzmann and Dr. Bödecker, have been voluminous; and while these have not been accorded full recognition, they have had a powerful influence in promoting investigation in the special lines of work in which he was most interested.

Dr. Abbott was born at Shapleigh, York County, Maine, on September 5, 1836. He was descended from one of the earliest Puritan settlers in that part of the country, the first American representative of the family having arrived in 1640. Originally the Abbotts belonged to one of the most ancient races in Europe, having existed in Sicily for many hundreds of years. Frank Abbott was educated at one of the Shapleigh schools, and when he was twenty years old became a student in the office of a dental surgeon in Oneida, New York. Afterwards he removed to Johnstown, New

York, where, with the exception of a few months in 1862, during which he served in the army, he practised as a dentist until 1863. While at Johnstown he married Miss Catherine Ann Cuyler, also a descendant of an old American family.

In 1863 he came to New York and attended the lectures at the medical department of the University of the City of New York, and took his M.D. degree. In 1866, upon the formation of the College of Dentistry, Dr. Abbott was appointed clinical instructor, and in 1868 he became a professor and one of the trustees of the same institution. In 1867 he established the infirmary connected with the college, and became its superintendent. In 1869 he was appointed dean, a place which he held until his death. Besides being dean at the Dental College, he was professor of dental histology, surgery, and therapeutics.

Dr. Abbott was a member of the New York County Medical Society, the Academy of Medicine, the American Dental Association, the New York Academy of Sciences, the American Numismatic and Archæological Society, the University Club, the Museum of Natural History, and a member, from its foundation, of the Metropolitan Museum of Art. He was also a Fellow of the American Geographical Society, and connected with numerous other bodies. He possessed a celebrated collection of rare prints dealing with American history, and was an enthusiastic book and picture collector.

His interest in dentistry was not confined to local organizations, but extended to the national associations, in which he was an ever-present and active worker. He was made president of the American Dental Association in 1887, and was also presiding officer for one year in the National Association of Dental Faculties. His work in the latter organization was always appreciated by his colleagues, and his active interest will be more and more missed as the years pass by.

The writer feels his death a personal loss, for, while often in antagonism, the warm friendship of many years has never been broken. His aim in life was to lead, and such men are the true pioneers who make paths for others to follow, and of such the world has too few.

He leaves a widow, two daughters, Mrs. Willet Coles Ely and Miss Katherine C. Abbott, and a son, Dr. Frank Abbott.

His funeral took place at Johnstown, New York.

